# Effects of home-based integrated sensory stimulation program to preterm infants on parents’ depression and anxiety: a randomized controlled trial

**DOI:** 10.1080/16549716.2025.2491848

**Published:** 2025-05-02

**Authors:** Wenjing Zheng, Rassamee Chotipanvithayakul, Thammasin Ingviya, Fang Guo

**Affiliations:** a Department of Pediatrics, The Second Affiliated Hospital of Kunming Medical University, Kunming, Yunnan, China; b Department of Epidemiology, Faculty of Medicine, Prince of Songkla University, Hat Yai, Songkhla, Thailand; c Research Center for Kids and Youth Development, Prince of Songkla University, Hat Yai, Songkhla, Thailand; d Department of Family and Preventive Medicine, Faculty of Medicine, Prince of Songkla University, Hat Yai, Songkhla, Thailand; e Department of Clinical Research and Medical Data Science, Faculty of Medicine, Prince of Songkla University, Hat Yai, Songkhla, Thailand; f Department of Neonatology, Affiliated Hospital of Kunming University of Science and Technology Clinical College, Kunming, Yunnan, China

**Keywords:** Multisensory stimulation, postpartum depression and anxiety, primary caregivers, premature baby, randomized clinical trial

## Abstract

**Background:**

Preterm parents face higher risks of postpartum depression and anxiety, affecting bonding and infant development. Sensory stimulation shows promise, but its long-term impact on parental mental health needs further study.

**Objectives:**

This study aimed to evaluate whether a home-based integrated sensory stimulation program, administered to preterm infants by their parents, could alleviate parental mental health issues and enhance maternal bonding and parenting competence.

**Methods:**

The program, including tactile, auditory, visual, gustatory, and olfactory stimuli, was assessed in a block-randomized controlled trial from November 2018 to January 2020. A total of 200 parents of preterm infants were recruited, and the intervention continued at home until the infants reached six months corrected age. Parents’ depression and anxiety were assessed using validated scales at baseline, and at first, third, and sixth month follow-ups.

**Results:**

The intervention group included 98 parents, and the control group comprised 102 parents. At the six-month follow-up, the intervention group demonstrated significant improvements in maternal depression, state anxiety, and trait anxiety compared to the control group. In the mixed linear model, the intervention was associated with reductions in maternal trait anxiety (d =-2.18; 95% CI: −4.30, −0.06), paternal trait anxiety (d =-3.37; 95% CI: −5.62, −1.11) and state anxiety (d =-4.63; 95% CI: −7.00, −2.26).

**Conclusion:**

The home-based integrated sensory stimulation program, when provided by parents to preterm infants, was effective in improving parents’ mental health and can serve as an alternative treatment for postpartum depression and anxiety in parents of preterm infants at home.

## Paper context

- **Main findings:** The home-based integrated sensory stimulation program significantly improved parental mental health, reducing depression and anxiety in both mothers and fathers of preterm infants.
- **Added knowledge:** The integrated sensory stimulation program can improve parents’ mental health for up to six months after discharge.
- **Global health impact for policy and action:** The program could be implemented without additional tools or effort, as feeding was already a part of daily routines.

## Background

Postpartum depression and anxiety affect 10–20% of mothers and 5% of fathers [1,2]. However, the prevalence is even higher among parents of preterm infants, with the rate of postpartum depression ranging from 40% to 65% and anxiety at 25% [3–5]. These parents face increased challenges, including higher levels of parenting stress, difficulties with feeding and sleeping patterns [6], and experience poorer interaction with their infants, which can lead to reduced synchrony, sensitivity, and emotional closeness [7–9]. These issues, in turn, impair maternal bonding [10]. In severe cases, suicide is responsible for 20% of maternal postpartum deaths [11].

Preterm infants often experience severe medical complications that require invasive treatments and prolonged hospitalization. They are also at higher risk for neurodevelopmental disorders, including speech and cognitive delays, compared to full-term infants [12]. Moreover, early preterm infants are particularly sensitive and vulnerable to environmental factors and the quality of nurturing care [13]. Consequently, insufficient parenting and poor bonding, often due to postpartum mental health issues, can exacerbate preterm infants’ health problems, including their developmental outcomes [14]. While pharmacological interventions have been proven to reduce postnatal psychiatric disorders, many breastfeeding mothers are reluctant to use them due to concerns about the transfer of drugs through breast milk and potential side effects for the infants [15]. Thus, there is a pressing need for effective non-medical interventions as alternative treatments.

Schore’s Right Brain Dual Corticolimbic-Autonomic Circuits theory [16] suggests that infants are susceptible to the quality of interaction with their caregivers. Sensory input from the visual, auditory, tactile, gustatory, and olfactory systems provided by parents can stimulate the infant’s brain and improve parents’ mental health. Kangaroo Care, a well-known program recommended by the WHO, also known as skin-to-skin care, involves holding newborns, especially preterm infants, skin-to-skin against a parent’s or caregiver’s chest [17]. This practice offers numerous benefits for both the infant and the caregiver. The physical contact and warmth of Kangaroo Care create a nurturing environment for the newborn, promoting physiological stability. Research has shown that it helps regulate the baby’s body temperature, heart rate, and breathing patterns, thereby reducing the risk of complications associated with premature birth [18]. Additionally, Kangaroo Care has been linked to improved weight gain and faster growth rates in premature infants, which are crucial for their overall development [19]. Beyond its physical benefits, Kangaroo Care strengthens emotional bonding between the infant and the caregiver. The close contact stimulates the release of oxytocin, often called the ‘love hormone’ in both the parent and the baby, fostering feelings of trust, security, and attachment. This emotional connection is essential for the infant’s social and emotional development and can have lasting positive effects on their well-being [20,21].

Furthermore, studies have highlighted the psychological benefits of Kangaroo Care for parents, including reduced anxiety levels and increased feelings of competence and emotional closeness with their baby [22]. However, some studies report contradictory findings regarding parents’ mental health. A correlation was observed in salivary cortisol levels reflecting stress between mothers and preterm infants in the skin-to-skin group. Still, no significant difference was found in parents’ cortisol levels compared to the standard care group. The authors pointed out that this may have been due to possible contamination of skin-to-skin practice in the control dyad group [23]. In addition, the emotional rollercoaster of having a premature or medically fragile infant can influence parental stress levels, regardless of whether Kangaroo Care is practiced [24]. The NICU (Neonatal Intensive Care Unit) environment itself can also be a source of stress for parents, with concerns about their infant’s health, medical procedures, and uncertainties about the future contributing to heightened anxiety levels [25]. Therefore, while Kangaroo Care may provide emotional benefits for parents, it may not completely alleviate the stress of having a baby in the NICU.

According to Schore, the brain’s right hemisphere is specialized in processing nonverbal emotional cues, regulating stress and arousal levels, and forming social bonds. The theory emphasizes the interconnectedness between the brain’s limbic system, which plays a key role in emotion regulation, and the role of multiple sensory inputs in promoting this process [16]. Therefore, interventions for preterm infants should prioritize multi-sensory stimulation to support their holistic development and well-being. But the impact of multiple sensory stimulation interventions on maternal mental health in the context of caring for preterm infants is also a critical consideration. One study in the United States employed four sensory stimulations, beginning with auditory, tactile, visual, and vestibular stimuli, conducted until infants reached a corrected age of two months. The study found a decrease in maternal distress, though the results were not statistically significant, likely due to an insufficient sample size [26]. As such, the effects of sensory stimulation on parents’ mental health warrant further investigation.

Most previous studies were conducted in NICU settings for a short duration and primarily focused on infant outcomes such as anthropometric measurements [27] and physiologic improvements [28]. However, the impact of the non-medical program on parents’ mental health is inadequate to establish clear recommendations or practice guidelines, mainly after they are discharged from the hospital. Furthermore, multiple sensory stimulation should be continuous, even after the infants are discharged, and evaluated over time.

This study is grounded in Schore’s Right Brain Dual Corticolimbic-Autonomic Circuits theory, which highlights the role of sensory input in regulating stress and arousal. It is the first randomized controlled trial to integrate visual, auditory, tactile, gustatory, and olfactory stimulation within the context of everyday activities in a home setting. The study aims to determine whether this program improves parental depression, anxiety, bonding, and parenting competence.

## Methods

### Study design and setting

A randomized controlled trial utilizing block randomization was conducted across three tertiary hospitals in Kunming, China, between November 2018 and January 2020. This method was employed to guarantee comparable sample sizes between the two groups. This study was registered with the Chinese Clinical Trial Registry. No substantial changes were made to the study design or methods after the commencement of the trial.

### Participants’ enrollment

Parents of preterm infants, including mothers or both fathers and mothers, were recruited if the infant was a singleton, had a gestational age of 28–36 weeks, and was discharged from the hospital without any serious illness. Both mothers and fathers were eligible if they were 18 years or older and legally capable of participating in complete civil activities. However, the dyad was excluded if the mother had a history of mental illness, was unable to care for their baby, or planned to relocate during the trial. A total of 200 subjects were required to detect a 15% reduction in mental health problems among parents. Parents were informed about the study and invited to participate on the NICU discharge day. If they agreed, written informed consent was obtained.

### Interventions

Families were randomly allocated in blocks of four or six to either the home-based integrated sensory stimulation intervention or standard care by a nurse independent of the study. The allocation process was concealed using opaque envelopes to mask group assignments. The intervention was based on the theory of Schore’s Right Brain Dual Corticolimbic-Autonomic Circuits, which posits that the right brain can be nurtured hierarchically by exteroceptive sensory input, aligning with Molapour’s concept [16,29].

#### Home-based integrated sensory intervention

The usual feeding practice consisted of tightly wrapping the baby in a blanket, restricted skin contact, and maintaining a quiet environment with minimal eye contact. In contrast, the intervention integrated five sensory systems, including tactile (gentle touch), auditory (voice), visual (eye contact), gustatory (breastmilk, formula milk, or other food), and olfactory (odor of caregiver). Parents were instructed on how to stimulate these systems through a 15-minute video clip, followed by practice with their infant under the guidance of a study investigator. The duration of stimulation varied between 10 and 30 minutes and took place during feeding time. Caregivers were advised to ensure their hands were clean and warm and that the baby’s diaper was changed before starting the stimulation. The caregiver held the baby closely so that the baby could receive their odor; stroked the exposed skin, such as the face and hands; captured the baby’s eyes and gave as much eye contact as possible; spoke to the baby in their local language or sang a song with a positive or loving feeling, such as a lullaby. The caregiver could choose which sensory system to use based on the baby’s behavioral cues. The researcher ensured that the primary caregiver fully understood the intervention and could administer it correctly before discharge from the hospital. The father, encouraged to follow the protocol, was informed that he could also assist with feeding when using a bottle.

#### Standard care

At discharge, all participants attended a routine health education session conducted by a well-trained nurse, using a prepared pamphlet, which lasted about 30 minutes. The nurse provided essential information about preterm infants, including potential health risks after discharge, how to recognize and manage abnormal symptoms, caring for a vulnerable baby at home, physical growth and neurodevelopmental milestones, as well as addressing potential parental mental health concerns and emphasizing the importance of a mother’s role in the infants’ development.

During the monthly follow-up visits in the first six months, all babies received vaccinations, growth and neurodevelopment surveillance, nutrition education, and vitamin A and D supplements. Thyroid function tests and funduscopic examinations were also performed if necessary. We organized separate visits for participants in individual rooms to prevent potential contamination.

#### Compliance

Compliance was defined as the intervention for at least 10 minutes per session, at least three times a day. It was assessed and recorded exclusively for the intervention group. Poor compliance is a failure to implement the intervention for the required 10 minutes in more than two of the seven daily sessions, exceeding 20% non-compliance.

Researchers conducted periodic assessments to evaluate parents’ adherence to the intervention and identify any barriers or challenges they may face. During each follow-up, parents in the intervention group were asked to describe how they implemented the intervention at home. If they could not completely follow the protocol, they were instructed to rewatch the video to refresh their understanding of the intervention details.

### Dependent and Independent variables

The study outcomes were assessed by standardized questionnaires to facilitate comparison with other studies. After parents signed the consent form, sociodemographic data were collected via face-to-face interviews, while the medical history of preterm infants and mothers was obtained from medical charts. Parents’ outcomes were assessed at baseline and again at infants’ corrected age of one, three, and six months. Both mothers and fathers reported their depression and anxiety levels at baseline and each follow-up point. Mothers reported their parenting competence at baseline and corrected age of one, three, and six months, as well as bonding at corrected age of three and six months.

#### Edinburgh Postnatal Depression Scale (EPDS)

The EPDS is a self-reported questionnaire comprising 10 items designed to assess symptoms of depression experienced by parents over the past seven days. Each item is scored from 0 to 3, with total scores ranging from 0 to 30. Values of 10 or higher were considered indicative of possible depression. The scale has been translated and tested in the Chinese population (*n* = 763), demonstrating good internal consistency, as reflected by a Cronbach’s alpha of 0.79. Validity studies have confirmed its effectiveness in screening for postnatal depression, with an overall content validity index of 0.93 [30].

#### Spielberger’s State and Trait Anxiety Inventory (STAI)

STAI is a widely used self-reported tool for assessing anxiety. It includes two subscales: state anxiety, which measures transient anxiety related to specific situations or experiences, and trait anxiety, which assesses relatively stable individual characteristics predisposing to anxiety. Each subscale consists of 20 items, with responses ranging from one (rarely) to four (almost always). Subscale scores range from 20 to 80, with higher scores indicating more significant anxiety. Values of 40 or higher on either subscale are indicative of a high level of anxiety. The scale has demonstrated strong internal consistency, with a Cronbach’s alpha of 0.84, suggesting high reliability, as tested in a Chinese population sample (*n* = 2,150). The STAI has been extensively validated across different populations and languages. The instrument has demonstrated robust validity in assessing anxiety in Chinese, featuring two stable factorial structures: the presence and absence of anxiety. It has shown good validity in assessing anxiety, with two stable factor structures [31].

#### Parent Sense of Competence Scale (PSOC)

The PSOC is a widely used self-report measure designed to assess parents’ perceptions of their parenting competence. It consists of items that capture two dimensions of parenting self-esteem: skill/knowledge and valuing/comfort. The version used in this study consists of 17 items. Parents rate each item on a six-point Likert scale, with higher scores reflecting a greater sense of parenting competence. The scale has demonstrated good internal consistency in 170 Chinese participants, as evidenced by a Cronbach’s alpha of 0.85. It also shows significant concurrent validity, with correlations of *r* = 0.60 with Rosenberg’s Self-Esteem Scale and *r* = −0.48 with the Edinburgh Postnatal Depression Scale [32].

#### Postpartum bonding questionnaire (PBQ)

Bonding between the dyad was measured using the Chinese version of the PBQ. The PBQ consists of 25 items separated into four sections, covering various aspects of the mother-infant relationship. Each item is rated on a scale from zero to five. For scoring purposes, items indicating a positive relationship are reverse-scored, meaning higher scores reflect worse bonding. The total score reflects the overall level of bonding, with higher total scores indicating poorer bonding. The PBQ has demonstrated good reliability and validity in measuring mother-infant bonding during the postpartum period in a Chinese sample of 62 women [33].

### Statistical methods

EpiData was used for data entry. R software version 4.0.5 was utilized for data analysis with epicalc, ggplot2, and lme4 packages.

The analyses of outcomes were based on the intention-to-treat principle. Descriptive statistics were calculated, including mean and standard deviations for continuous variables (e.g. mental health scores) and the frequencies and percentages for categorical variables. Paired sample t-tests and McNemar’s chi-square tests were used to assess differences between pre- and post-intervention at each follow-up stage. The effect of the intervention throughout the entire follow-up period was estimated using a linear mixed-effects model. A *p*-value of < 0.05 was considered the threshold for statistical significance.

## Results

**Figure 1.**
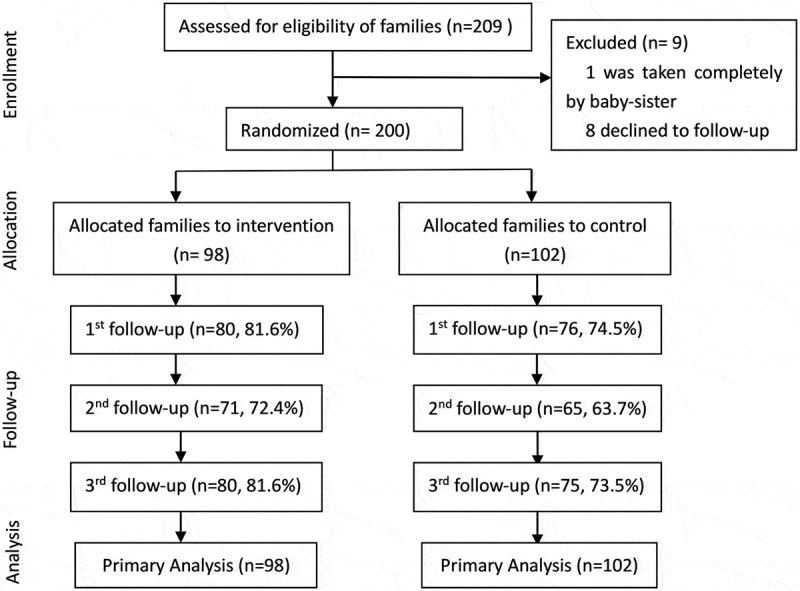
Flow chart of participants’ number during enrollment, allocation, follow-up, and analysis.

After excluding nine families (one with unavailable parents and eight with follow-up at other hospitals), 200 families were included in the analysis; 98 were in the intervention group, and 102 were in the standard care group. The follow-up rates over the six months ranged from 72.4% to 81.6% in the intervention group and from 63.7% to 74.5% in the control group (Figure 1).

### Baseline Characteristics of the Parents and Preterm Infants

Table 1 presents the demographic characteristics of the parents. The average age of both mothers and fathers was 30 years. Approximately half of the parents had education beyond high school, held skilled jobs, and had more than one child. About one-third of parents reported an average monthly income of over 10,000 yuan (USD 1576.69). Among the 165 mothers, one-third reported possible depression, state anxiety, and trait anxiety, respectively. Among fathers, 15.0% reported possible depression, 33.0% reported state anxiety, and 29.5% reported trait anxiety. Notably, state anxiety (47.3%) and trait anxiety (40.7%) were significantly higher among fathers in the control group compared to those in the intervention group.

**Table 1.** Baseline characteristics of the intervention and control groups.

	Total (*n* = 200) n (%)	Intervention (*n* = 98) n (%)	Control (*n* = 102) n (%)
Parents’ characteristics			
Mother’s age (years), mean (SD)	30.1 (4.8)	30.2 (4.9)	30.0 (4.8)
Mother’s education			
Under junior middle school	53 (26.5)	20 (20.4)	33 (32.4)
High school	47 (23.5)	24 (24.5)	23 (22.5)
Higher than high school	100 (50.0)	54 (55.1)	46 (45.1)
Mother’s occupation			
Skilled worker	81 (40.5)	42 (42.9)	39 (38.2)
Semi-skilled worker	16 (8.0)	9 (9.2)	7 (6.9)
Non-skilled worker	103 (51.5)	47 (48.0)	56 (54.9)
Father’s age (years), mean (SD)	32.3 (5.7)	32.5 (5.8)	32.0 (5.5)
Father’s education			
Under junior middle school	53 (26.5)	20 (20.4)	33 (32.4)
High school	44 (22.0)	23 (23.5)	21 (20.6)
Higher than high school	103 (51.5)	55 (56.1)	48 (47.1)
Father’s occupation			
Skilled worker	102 (51.0)	56 (57.1)	46 (45.1)
Semi-skilled worker	19 (9.5)	8 (8.2)	11 (10.8)
Non-skilled worker	79 (39.5)	34 (34.7)	45 (44.1)
Average family monthly income (yuan)			
<5,000	55 (27.5)	25 (25.5)	30 (29.4)
5,000–10,000	84 (42.0)	39 (39.8)	45 (44.1)
>10,000	61 (30.5)	34 (34.7)	27 (26.5)
Having sibling	99 (49.5)	46 (46.9)	53 (52.0)
Planned pregnancy	179 (89.5)	92 (93.9)	87 (85.3)
Family support	176 (88.0)	87 (88.8)	89 (87.3)
High-risk pregnancy	138 (69.0)	71 (72.4)	67 (65.7)
Infants’ characteristics			
Gender			
Female	90 (45.0)	42 (42.9)	48 (47.1)
Male	110 (55.0)	56 (57.1)	54 (52.9)
Gestational age (weeks), mean (SD) Delivery phase	34.6 (1.8)	34.5 (1.8)	34.6 (1.8)
Early and Moderate (28–33 weeks)	61 (30.5)	31 (31.6)	30 (29.4)
Late (34–36 weeks)	139 (69.5)	67 (68.4)	72 (70.6)
Birth weight (grams)			
<2,500	135 (67.5)	63 (64.3)	72 (70.6)
2,500–4,000	65 (32.5)	35 (35.7)	30 (29.4)
Mechanical ventilation			
No	144 (72.0)	68 (76.5)	76 (74.5)
Without intubation	44 (22.0)	23 (23.5)	21 (20.6)
With intubation	12 (6.0)	7 (7.1)	5 (4.9)
Sepsis	5 (2.5)	3 (3.1)	2 (2.0)
Home oxygen required	13 (6.5)	5 (5.1)	8 (7.8)
Length of stay (days), mean (SD)	14.1 (11.0)	13.8 (10.7)	14.6 (11.2)

Half of the infants were male, one-third were early and moderate preterm (28–33 weeks gestational age), and two-thirds were late preterm (34–36 weeks gestational age). A comparison of baseline characteristics, including gestational age, delivery mode, gender, birth weight, and medical complications, revealed no significant differences between the intervention and control groups (Table 1).

### Comparing the mental health, parenting competence, and bonding between the intervention and control groups during six months

**Figure 2.**
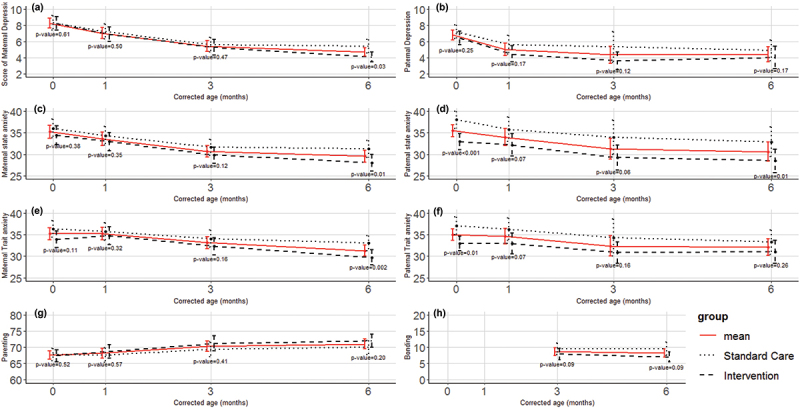
Comparison of parents’ mental health scores, parenting, and bonding between the intervention and control groups. *(a). maternal depression; (b). paternal depression; (c). maternal state anxiety; (d). paternal state anxiety; (e). maternal trait anxiety; (f). Paternal trait anxiety; (g). parenting competence; (h). bonding. P-values at each time point compare the intervention and control groups.

Over the six months of intervention, parents reported compliance rates of 52%, 71%, and 66% at the first, second, and third follow-ups, respectively. Figure 2 shows improvements in both mothers’ and fathers’ depression, anxiety, and parenting competence, particularly in the first three months. At the six-month follow-up, maternal depression, state and trait anxiety scores, and paternal state anxiety were significantly lower in the intervention group compared to the control group. Bonding slightly improved from three to six months, but this change was not significantly different from baseline.

Table 2 shows that maternal depression and anxiety statistically decreased from baseline to six months in the intervention group, while maternal depression and state anxiety, as well as paternal depression and state anxiety, statistically decreased from baseline to six months in the control group. The rate of reduction in maternal depression and anxiety in the intervention group was approximately two to four times higher than in the control group. However, the reduction rates for paternal depression and anxiety were similar between the intervention and control groups. After applying the Bonferroni correction, the results remained significant, except for paternal depression and trait anxiety.

**Table 2.** Comparison of mothers’ (*n* = 165) and fathers’ (*n* = 182) mental health problems between the intervention and the control groups.

	Intervention n (%)	Control n (%)	P
Maternal depression			
Baseline	28 (35.4)^a^	29 (33.7)^w^	0.95
1-month follow-up	20 (25.0)	22 (29.3)	0.67
3-month follow-up	8 (11.4)	11 (17.2)	0.48
6-month follow-up	3 (3.8)^a^	10 (13.7)^w^	0.06
Maternal trait anxiety			
Baseline	24 (30.4)^b^	30 (34.9)	0.65
1-month follow-up	25 (31.2)	30 (40.0)	0.33
3-month follow-up	15 (21.4)	18 (28.1)	0.49
6-month follow-up	6 (7.6)^b^	18 (24.7)	0.01
Maternal state anxiety			
Baseline	25 (31.6)^c^	31 (36.0)^x^	0.67
1-month follow-up	19 (23.8)	27 (36.0)	0.14
3-month follow-up	9 (12.9)	11 (17.2)	0.65
6-month follow-up	6 (7.6)^c^	12 (16.4)^x^	0.15
Paternal depression			
Baseline	15 (16.5)	21 (23.1)^y^	0.35
1-month follow-up	4 (10.5)	7 (17.9)	0.55
3-month follow-up	0 (0)	4 (22.2)	0.03
6-month follow-up	2 (8.0)	0 (0)^y^	0.49
Paternal trait anxiety			
Baseline	23 (25.3)	37 (40.7)	0.04
1-month follow-up	8 (21.1)	15 (38.5)	0.16
3-month follow-up	2 (8.7)	5 (27.8)	0.21
6-month follow-up	3 (12.0)	6 (26.1)	0.28
Paternal state anxiety			
Baseline	23 (25.3)	43 (47.3)^z^	0.003
1-month follow-up	9 (23.7)	13 (33.3)	0.49
3-month follow-up	2 (8.7)	4 (22.2)	0.38
6-month follow-up	2 (8.0)	4 (17.4)^z^	0.41

*Items with the same superscript (a-a, b-b, c-c, w-w, x-x, y-y, z-z) indicate that the outcome significantly decreased from baseline to 6-month follow-up.

The mixed model results, presented in Table 3, showed a significant decrease in parents’ mental health problems over time. However, the decline was more pronounced in the intervention group, particularly for maternal trait anxiety (*d* =-2.18; 95% CI: −4.30, −0.06, *p* = 0.04) vs. (*d* =-1.28; 95% CI: −1.68, −0.88, *p* < 0.001), paternal state anxiety (*d* =-4.63; 95% CI: −7.00, −2.26, *p* < 0.001) vs. (*d* = −1.70; 95% CI: −2.35, −1.04, *p* < 0.001), and paternal trait anxiety (*d* = −3.37; 95% CI: −5.62, −1.11, *p* = 0.03) vs. (*d* = −0.92; 95% CI-1.51, −0.32, *p* = 0.002).

**Table 3.** Results of the mixed model for parents’ mental health.

	Time		Intervention	
	Coefficient (95% CI)	P-value	Coefficient (95% CI)	P-value
Mother				
- Depression	−1.09 (−1.28, −0.91)	<0.001	−0.44 (−1.36, 0.47)	0.34
- State anxiety	−1.77 (−2.20, −1.35)	<0.001	−1.91 (−4.13, 0.31)	0.09
- Trait anxiety	−1.28 (−1.68, −0.88)	<0.001	−2.18 (−4.30, −0.06)	0.04
Father				
- Depression	−0.66 (−0.91, −0.42)	<0.001	−0.86 (−1.80, 0.08)	0.07
- State anxiety	−1.70 (−2.35, −1.04)	<0.001	−4.63 (−7.00, −2.26)	<0.001
- Trait anxiety	−0.92 (−1.51, −0.32)	0.002	−3.37 (−5.62, −1.11)	0.003

CI: Confidence interval.

## Discussion

This study found that approximately 30% of mothers experienced depression and 30% experienced anxiety. In comparison, 15% of fathers had depression and 30% had anxiety when the infants were deemed stable enough for discharge from NICU. At the six-month follow-up, the intervention group demonstrated significant reductions in state anxiety, paternal trait anxiety, and maternal trait anxiety scores compared to the control group.

Consistently, other studies among parents of preterm infants have reported that 29.4% of mothers experienced depression and 26.5% had anxiety, while 15% of fathers reported depression and 25% experienced anxiety [3,34]. A multi-country study conducted across 15 developed countries found postpartum depression rates of 25.3% among mothers and 8.3% among fathers of preterm infants, even after receiving a family-centered care program during their NICU stay [35]. This may be because infants born unexpectedly early leave parents unprepared for feeding and care, which can increase maternal stress and anxiety [36]. The impact of parents’ mental health on infant caregiving has been shown to result in lower interaction, reduced sensitivity, and inadequate caregiving [37].

Most parents are unaware of their mental health problems or may avoid seeking professional help due to stigmatization and systemic barriers, including service and policy limitations that prevent adequate care [38]. Although medical treatments have been proven to be effective for postpartum depression, some mothers decline medication due to concerns that the drugs may transfer to their infants through breastfeeding [39]. Non-medical interventions, such as peer support from hospital to home, lifestyle training, and smartphone-based psychoeducation from prenatal to postnatal period, have also been beneficial for parents’ mental health at four to six weeks postpartum [40–42]. However, these programs primarily focus on reducing depression and anxiety, and may not address bonding or infant development, particularly the long-term outcomes after discharge. In contrast, our program not only supports parents’ mental health but also promotes the development of preterm infants [43].

Our home-based integrated sensory stimulation program showed significant improvements in parents’ mental health, including reductions in depression and anxiety, as well as better bonding with their babies compared to standard care. It reduced the proportion of depression and anxiety among mothers from 30% to 35% to 5% to 7% over six months. Although a previous longitudinal study [44] observed that parents’ depression, anxiety, and parenting stress decreased over time without any intervention, another study found that the proportion of parents with these problems remained high (15%) after six months [45]. Our intervention reduced maternal trait anxiety and paternal anxiety (state and trait) two to four times more than the control group. Additionally, the intervention produced a more significant effect size than the natural passage of time, potentially accelerating the recovery of parents’ mental health and enabling them to provide better care for their babies, especially in the baby’s first year of life.

Our program was based on the theory that the mother-infant interaction through sensory stimulation could enhance attachment. Through reciprocal affection exchanges, mothers and infants can resonate with each other, with mothers adjusting their arousal state according to their infants’ arousal levels [16]. A meta-analysis suggested that mothers who had a natural delivery and practiced skin-to-skin care experienced lower anxiety [46]. Mothers who provided scent exchange, touch, and holding with their preterm infants reported reduced depression and anxiety [47]. Kangaroo care can also improve attachment between mother and infant and reduce fathers’ anxiety levels [48,49].

Bonding and parenting competence take time to develop. Most previous studies have examined the effects of sensory stimulation over a short period (three months or less) [50,51], which may not be sufficient for adequately assessing parent-child bonding and parenting competence. Our study extended the follow-up period to six months. Although statistical differences were not observed, we found a positive trend in bonding and parenting competence over this time.

Feeding provides an appropriate opportunity to promote mother-child interaction. However, one study showed that mothers had minimal or inconsistent synchrony with their babies during feeding, such as touch and gaze [52]. In today’s digital age, mothers frequently use devices during feeding, which can reduce mother-infant bonding [53]. To promote interaction between parents and their babies, our program is feasible and easily incorporated into daily life during feeding time. We recommend all parents, especially those with preterm infants, practice this sensory integration program during feeding.

The intervention demonstrates significant implications for improving parental depression and anxiety, particularly for parents of preterm infants. The home-based integrated sensory stimulation program provides better care and support for their infants, particularly during the first year. Therefore, the program not only reduces depression and anxiety but also enhances bonding and parenting competence over six months, addressing a gap in previous interventions. Additionally, its integration into daily routines like feeding promotes more consistent mother-infant interaction, making it feasible and effective for improving long-term developmental outcomes for preterm infants.

The randomized controlled trial design is a primary strength of the study, providing the strongest evidence to assess the program’s effectiveness. An intention-to-treat analysis was used to ensure the effects of randomization. Adequate sample size and a high follow-up rate provided sufficient study power to detect any impact of the intervention. Following best practices for reporting randomized controlled trials (RCTs), this study adheres to the CONSORT (Consolidated Standards of Reporting Trials) guidelines [54].

One limitation of the study is the low sample size of fathers compared to mothers, which suggests that caution should be exercised in interpreting and utilizing the findings concerning the fathers. Another limitation is the lack of blinding, which could lead to information bias. Moreover, the study mainly relied on self-report measures for assessing adherence to the intervention, particularly for complex interactions like sensory stimulation with infants. This introduces potential risks, as inaccurate reporting could either overestimate or underestimate adherence levels, leading to bias. This discrepancy between reported adherence and actual implementation could impact the results. Additionally, a low level of adherence raises concerns about the fidelity of the intervention delivery and its potential impact on the study outcomes. Significant results can still emerge despite low adherence. However, it’s essential to interpret these findings cautiously and consider the possible effects of low adherence on the validity and generalizability of the results. Thus, further studies should address these challenges to strengthen the evidence. Lastly, the home-based delivery of the intervention poses another limitation, as it may have restricted the ability to make real-time adjustments. If delivered in a hospital setting, immediate corrective actions could have been more easily implemented, enabling better oversight and personalized adaptations to individual needs.

In many cultures, there may be stigma associated with mental health issues, particularly postpartum depression and anxiety. This stigma can prevent parents from seeking professional help or participating in interventions aimed at improving mental health outcomes. Additionally, cultural norms and expectations regarding parental roles and responsibilities may vary, affecting the acceptability and feasibility of specific interventions. The effectiveness of sensory stimulation programs may also vary across cultures due to differences in sensory stimulation programs in parenting styles, family dynamics, and social support networks. Cultural values and beliefs regarding touch, physical contact, and infant care practices may influence the acceptability and implementation of sensory stimulation interventions. Furthermore, access to resources and healthcare services may differ between cultures, affecting the availability and implementation of sensory stimulation interventions. Socioeconomic factors, such as income level, education, and access to healthcare, can also impact the likelihood of parents engaging in sensory stimulation programs. While our study provides valuable insights into the effectiveness of a home-based integrated sensory stimulation program in improving parental depression, anxiety, and bonding with preterm infants, it is essential to acknowledge the limitations of generalizability. Cultural differences across diverse populations may influence the applicability and effectiveness of the intervention. Therefore, future research should explore the cultural relevance of sensory stimulation programs and consider adaptations to ensure their effectiveness in various cultural contexts.

## Conclusion

Postpartum mental health is a common problem, especially among parents of preterm infants. Our home-based integrated sensory stimulation program could improve parents’ depression and anxiety after six months. The program can be a viable non-pharmacological intervention. We recommend that parents of preterm infants practice the program for at least six months to reduce depression and anxiety.

## Data Availability

Complete datasets and other study materials can be obtained from the corresponding author upon reasonable request.
